# Remarks on a Recently Discovered Mineral Spring at Portage, Mich.

**Published:** 1870-11

**Authors:** Edward Clapham

**Affiliations:** Kalamazoo, Mich.


ARTICLE IV.—*Remarks on a Recently Discovered Mineral Spring at Portage, Kalamazoo County, Mich.;* together with the Qualitative Analysis thereof, in comparison with some European Springs of Celebrity. By EDWARD CLAPHAM, M.D., L.R.C.S., etc., Kalamazoo, Mich.

During the past few months there has been quite a temporary *furore* created in this section of our good State of Michigan, by the discovery of numerous mineral springs, endowed with high magnetic properties, and invested with wondrous curative powers. Perhaps nothing, since the discovery of Petroleum, has so similarly started people hunting for magnetic springs all over their lands, round about here, in the hope of realizing largely on their property should such a treasure haply be found. Various have been the samples of water brought me for an opinion or an analysis, but in few cases have they proved worthy of a second consideration. The public have become satiated alike with water and the vaunted efficacy thereof, which each spring was said to possess. But since this magnetic-spring fever has abated somewhat, it is proper to speak of one which has hitherto not been pushed into notice, other than by its merits as a remedial agency.

The spring in question is on the property of Mr. Durkee, of Portage, Mich, and issues from a hillside, sloping gently down to the bank of a small stream, being pleasantly situated amid a grove of handsome shade trees. Bath houses of a temporary nature have been erected close to the spot whence the water issues, so that the full benefit of the same is amply secured to the invalids.

The *external* application of this water appears to be decidedly sedative, from accounts I have received of it from those who have derived benefit, and may be due to the pretty large quantity of carbonic acid dissolved in the water, and also the salts held in solution by the same gas. The late Sir James Y. Simpson was the first to demonstrate the decided sedative action of carbonic acid gas, applied to various parts of the body, chiefly mucous or abraded surfaces, however; but there is no reason why a bath of highly carbonated water should not exert a very happy influence over cutaneous surfaces. Hence, to this agent ($CO_2$) we may attribute, I suppose, the relief experienced by many who have made use of the Durkee Spring in cases of rheumatic affections.

In its nature and composition this spring more closely resembles that noted one of "Spa," in Belgium, as will be seen at once from the respective analyses given below for comparison; and by a rather strange coincidence, prior to my acquainting Mr. Durkee with the result of my analysis of his spring, it had been remarked to him by one who had made use of it, that "it more closely resembled the celebrated European medicinal spring baths, than any he had seen since his return from Europe."

That this spring possesses some curative power, there can be but little doubt, as a glance at its composition shows, but as medical men, remembering the effects of mind on matter, and especially the value of free ablutions to bodies which, perhaps, have been neglected in this particular, we must accept the *marvellous* with the usual number of grains of salt.

The existence of an appreciable quantity of Bicarbonate Soda in this spring, entitles it, however, to some little consideration on our part, as also the large amount of Carbonate Protoxide of Iron, held in perfect solution, and therefore in a most agreeable and assimilable form perhaps.

These two bodies form the chief peculiarity of the water as medicinal agents, nor have they been found present as regards the Carbonate Soda, or in as large quantities as regards the Iron, in any of the springs around here, which have been so highly extolled and quite a fuss made of them.

I regret that pressure of professional business and the lack of a pair of fine analytical balances just now, have prevented me giving your readers a full quantitative analysis of this rather interesting spring, for there is no doubt that under due and proper medical advice some benefit may be derived in chronic derangements of the skin, stomach, liver, kidneys and bowels, conjoined with various hygienic requirements which can be secured at the farm house near the spring, which is only nine miles from Kalamazoo, and conveniently reached twice daily by the Lake Shore and Michigan Southern R. R.

*Analysis of the Durkee Spring.*

Temperature at which the water issues from the earth, 50° Fah.
Specific Gravity, 1·005 at 60° Fah.

Carbonic Acid, *free*, considerable, but undetermined.
Sulphureted Hydrogen, *free*, traces.
*Bicarbonate Soda.*
Bicarbonate Magnesia.
Carbonate Lime.
Chloride Sodium.
*Carbonate of Protoxide Iron.*
Sulphate Lime.
Alumina.
Silica.
Organic matters.

Total number of grains of solid matter held in solution by one gallon of this water at 50° Fah., 26·25.

The quantity of *free* $CO_2$ is not unusually large, but considerable exists in a nascent form, holding in solution, at ordinary temperature, the saline and mineral constituents, NaO, MgO, CaO, and FeO.

*Qualitative Analysis of the Celebrated Spring of "Spa," Belgium.*

Temperature, 50° Fah.
*Carbonate Soda.*
Carbonate Magnesia.
Carbonate Lime.
Chloride Sodium.
*Protoxide Iron.*
Free Carbonic Acid Gas.

It will be observed that these spring waters are very similar and coincide in all important particulars, being of a saline and chalybeate nature, and furthermore it may be cited that if we except the difference of Oxyde of Iron and a few degrees of temperature, the Durkee Spring closely resembles the celebrated Thermal Spring, of Plombiers (France) a favorite and frequent place of resort of the late Emperor Napoleon the third, whose chronic malady found relief thereat.

By slightly raising the temperature by artificial means, Mr. Durkee is enabled to furnish a very pleasant and efficacious bath, equal to that of Plombiers, the analysis of which is given herewith.

*Qualitative Analysis of the Spring at Plombiers, France.*

Temperature, 90° Fah., (variable, however.)
*Carbonate Soda.*
Carbonate Lime.
Chloride Sodium.
Sulphate Soda.
Silica, and organic matters.

From personal experience of the Durkee Spring, I can only remark, in conclusion, that it is exceedingly pleasant and refreshing. Large quantities can be swallowed without the usual feeling of oppression or distension. I purposely pass over any allusion to the magnetic properties (so called) which the spring may have been found to possess, as I attach but very little importance to the phenomena of rendering knives magnetic by immersion in these springs as viewed in a medical light.

I have my own theory as to the frequent discovery of these pseudo-magnetic springs, which is, that owing to a large extent of bog iron ore, stretching away from Canada down as far as our peninsula of Michigan, and this is well known to possess magnetic properties (having specimens in my own mineralogical cabinet) there is no wonder at the development of magnetic phenomena, or of the large quantity of iron found, in these springs at their source. Capital has been made (or attempted) of this imponderable agency in connection with the various springs, but it is somewhat remarkable that in curative results, the Durkee Spring, itself possessing scarcely any magnetic properties, has wrought more than any others which vaunt their magnetism.

This spring water is decidedly diuretic and aperient, also perceptibly detersive in its action on the skin as well as in cleansing textile fabrics.

From persons of veracity I have heard many accounts of its sedative influence upon limbs as well as other parts of the body generally, which being undeniable can only be referred to the presence of appreciable quantities of alkali (NaO) in a highly carbonated form ($=^2 CO_2$) and on this account, therefore, externally, in conjunction with its *aperient* and *anti-lithic* effects internally, I was led to believe that my professional brethren might wish to learn some particulars as to the relative character and composition of this rather interesting spring, which may haply attain celebrity some day, but which at present may only excite the action of "musculi risorii, Santorini nostri," with the accompanying remark, "bibat qui vult."

P. S. The analyses of the "Otsego," "Eaton Rapids" and "Collins" springs having already been made public, I have not deemed it necessary to reproduce them here, as comparison, if ever odious, would be so in this case especially.

